# Revisiting Rossion and Pourtois with new ratings for automated complexity, familiarity, beauty, and encounter

**DOI:** 10.3758/s13428-016-0808-z

**Published:** 2016-10-03

**Authors:** Alex Forsythe, Nichola Street, Mai Helmy

**Affiliations:** 1 Department of Psychology, University of Liverpool, Liverpool, UK; 2 Department of Psychology, Staffordshire University, Stoke-on-Trent, UK; 3 Department of Psychology, Menoufia University, Al Minufya, Egypt

**Keywords:** Norms, Complexity, Familiarity, Pictures

## Abstract

Differences between norm ratings collected when participants are asked to consider more than one picture characteristic are contrasted with the traditional methodological approaches of collecting ratings separately for image constructs. We present data that suggest that reporting normative data, based on methodological procedures that ask participants to consider multiple image constructs simultaneously, could potentially confounded norm data. We provide data for two new image constructs, beauty and the extent to which participants encountered the stimuli in their everyday lives. Analysis of this data suggests that familiarity and encounter are tapping different image constructs. The extent to which an observer encounters an object predicts human judgments of visual complexity. Encountering an image was also found to be an important predictor of beauty, but familiarity with that image was not. Taken together, these results suggest that continuing to collect complexity measures from human judgments is a pointless exercise. Automated measures are more reliable and valid measures, which are demonstrated here as predicting human preferences.

## Introduction

Subjective ratings have been an established method by which to produce normative data for language and picture research (see Proctor & Vu, 1999, for a review). Paivio and his colleagues were one of the first to obtain normative ratings of concreteness, imagery, and meaningfulness in what was to become one of the best-known sets of normative ratings for the imageability, concreteness, and meaningfulness of words (Paivio, Yuille, & Madigan, 1968). Their motivation for obtaining ratings was the lack of appropriate normative data for word characteristics that they wished to investigate in the course of their research. Prior research had sometimes relied on “*unspecified judgements by the experimenter alone*” (p2). Since Proctor and Vu, further norms have been reported for not only words but also icons and symbols (Forsythe et al., 2003, 2008; McDougall et al., 2000, 1999), most extensively for picture sets (e.g., Alario et al., 2004; Barry, Morrison, & Ellis, 1997; Bates et al., 2003; Bogka et al., 2003; Bonin, Barry, Méot, & Chalard, 2004; Bonin, Chalard, Méot, & Fayol, 2002; Cuetos, Ellis, & Alvarez, 1999; Dell’Acqua, Lotto, & Job, 2000; Dimitropoulou et al., 2009; Ellis & Morrison, 1998; Lloyd-Jones & Nettlemill, 2007; Morrison & Gibbons, 2006; Morrison, Hirsh, & Duggan, 2003; Rossion & Pourtois, 2004; Snodgrass & Yuditsky, 1996; Snodgrass & Vanderwart, 1980; Vitkovitch & Tyrrell, 1995; Weekes, Shu, Hao, Liu, & Tan, 2007; Zevin & Seidenberg, 2002) and recently for art (Forsythe et al., 2011).

Following on from the initial classic work by Snodgrass and Vanderwart (1980), Rossion and Pourtois were interested in examining visual complexity. Snodgrass and Vanderwart suggested how, in episodic memory tasks, complexity is likely to influence stimulus recognition. The extra detail depicted in an object may give an image added novelty, and this novelty may slow the recognition process. The authors felt it likely that increased complexity would influence the speed at which pictures are categorized, man-made objects being simpler would be categorized most quickly, and naturalistic complex images, such as insects or trees, would be categorized more slowly. Some categorical reaction time advantage has been reported – natural categories tend to be responded to more quickly than other natural categories – although this seemed to be mainly a function of diagnostic color, for example such as fruits/vegetables versus animals, rather than a function of complexity (Rossion & Pourtois, 2004).

Other researchers have reported this variability in complexity effects. Some have suggested that increased complexity can enhance performance (Biederman, 1987; Lloyd-Jones & Luckhurst, 2002), other have argued that visual complexity increases processing time (and hence naming time) at, or before, the stage of object recognition (Alario et al., 2004; Ellis & Morrison, 1998; Humphreys, Riddoch, & Quinlan, 1988). One reason for variations in complexity effects is possibly explained by the way in which researchers have attempted to quantify what is complex. The metrics used to determine complexity within images differed between researchers and in some cases complexity was confounded with other variables such as concreteness (McDougall et al., 1999; and see Forsythe et al., 2008). As such, researchers have sought to find ways to standardize the measurement of complexity (Forsythe et al., 2008) or, as previously mentioned, to develop sets of standardized images for use in testing.

### Measuring complexity

The study of visual complexity emerged from the empiricist tradition. The tradition is based on the premise that people make poor intuitive judges, and understanding could only be advanced through quantification in controlled laboratory settings. When unusual, unexplainable results emerged, Gestalt psychology developed to explain them. The Gestaltists set out to understand the processes of perception, not through the meticulous analysis of patches of light, shape, and color, but through an analysis of the whole, configuration, or form (Hochberg, 1986). Their philosophy was that sensations are not elementary experiences; we “see” shape and form regardless of where the image falls on the retina or what neurons process the various image components. What was important was constancy. One such law generated through the Gestalt movement was Prägnanz. The Prägnanz principle contends that the forms that are actually experienced take on the most parsimonious or “best” arrangement possible in given circumstances. In other words, of all the possible perceptual experiences to which a particular stimulus could give rise, the one most closely fitting to the concept of “good” will be experienced.

Koffka (1935) proposed that the term “good” means symmetrical, simple, organized, and regular. In his study of psychological organization, Kofka explained the tendency to create psychologically simple order patterns from a wide range of perceptual stimuli. This early study of “simplicity” evolved into the study of “complexity,” with theorists attempting to re-write the Gestalt law of simplicity within a more formal framework (Attneave, 1954; Attneave & Arnoult, 1956; Hochberg & Brooks, 1960). Both Hochberg and Attneave acknowledged that shape was a multidimensional variable that would vary with the complexity of an image, with Hochberg and Brooks going on to developed what was the first semi-automated measure of image complexity, arguing that relying solely on human judgments of complexity would mean that they had no way of predicting just how complex or simple an image would appear.

### Later approaches to visual complexity

Following the work of Attneave and Arnold, complexity has received less attention, in part because no universally acceptable metric existed. Those measures that had been developed historically were not particularly well supported within a theoretical framework (Johnson et al., 1996). For example, Geiselman et al. (1982) developed an index of discriminability between graphic symbols and identified nine “primitive” attributes, e.g., numbers of straight lines, arcs, quasi-angles, and blackened-in elements. Symbols selected for high discriminability using this metric were responded to faster than those with lower discriminability. Garcia et al. (1994) also sought to count the number of primitive attributes in icons and signs in order to determine how concrete, or pictorial, the icon was. Unfortunately for the authors, this proved to be a much better measure of visual complexity than concreteness (see McDougall et al., 1999). Garcia et al. reported that icons that are pictorially similar to their real-world counterparts are more likely to be judged as complex. This has been found not to be the case: complexity is more closely related to search efficacy (Dell’Acqua et al., 2000). A more valid and reliable measure of complexity would enable researchers to determine more accurately the effects of extra detail and intricacy on performance.

Forsythe et al. (2003, 2008) tested several automated measures of complexity based on measurements of the changes in image intensity (Beck et al., 1991; Harwerth & Levi, 1978; Sutter et al., 1989; Vassilev & Mitov, 1976). More recent work (Forsythe et al., 2011), examined the relationship between information processing models suggested by Shannon and Weaver (1949) and image compression as a measure of visual complexity. When images contain few elements or are homogenous in design, there are few message alternatives and as such the file string contains mostly numbers to be repeated. A more complex picture will have a less predictable number string. These measures seem to have good reliability when compared with human judgments of visual complexity (Forsythe et al., 2008, 2011) and have contributed towards a general disposition towards the development complexity metrics in the field (Machado, Romero, Nadal, Santos, Correia, & Carballal, 2015; Marin & Leder, 2013).

### Familiarity and complexity

**Fig. 1:**
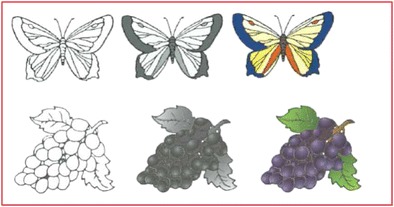
Examples of the Rossion and Pourtois images

The idea that observers make poor judges of visual complexity is important when considering the conventions of data collection. Rossion and Pourtois (2004) collected ratings on a number of stimulus variables (familiarity, concreteness, complexity, etc.,) for new versions of pictures in the style of Snodgrass and Vanderwart (1980). Previously only line drawing versions of these images had existed, and Rossion and Pourtois created versions in outline, color, and gray-scale (Fig. 1).

Historically, groups of raters had been employed in the collection of picture norms, with each group being asked only to consider one image construct (see Proctor & Vu, 1999 for a review). Rossion and Pourtois did not follow this tradition; rather groups of 20 subjects performed both the complexity *and* familiarity tasks. This could be problematic as Forsythe et al. provide experimental evidence that when observers are made more familiar with objects with no semantic content (i.e., nonsense shapes) they begin to rate those objects as less complex than they actually are, suggesting that the complexity data collected from Rossion and Pourtois could be confounded with familiarity judgments. This confound would somewhat explain the large correlations between complexity and familiarity judgments reported in the Rossion and Pourtois data set; correlations which are atypical in most other picture data sets.

Of course, if familiarity is a part of the construct of complexity, then this is what researchers and designers may need to take into consideration rather than simply arriving at the best context-free measure of visual complexity. The collection of ratings that contain both a complexity and familiarity component is not necessarily inherently bad and removing familiarity effects is not an advantage in its own right, it depends on what one wants. With this caveat in mind, we present a contrast analysis with the Rossion and Pourtois data set for the variables familiarity and complexity, with new ratings collected by separate groups of observers. We also provide for researchers automated data for the variable visual complexity, which is based on the Gif metric reported by Forsythe et al. (2011).

### Beauty and complexity

**Fig. 2:**
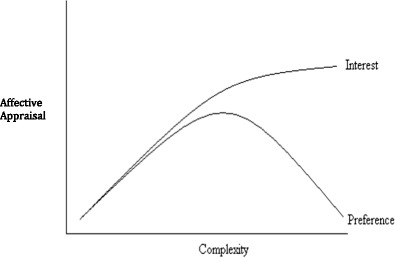
Berlyne (1971), the effect of complexity on preference and interest

In the study of beauty, Berlyne’s (1971) curvilinear relationship with visual complexity has received the most attention. Berlyne argued that complexity increases linearly with preference until an optimum level of visual arousal is reached (Fig. 2). At this point further increases in complexity would elicit a downturn in arousal and preference would decrease. In other words, when visual stimuli are of low complexity (i.e., simple), preference and judgments of beauty will also be low. People will seek to maintain a level of arousal that supports their preferred level of stimulation. Individuals who are highly aroused will seek out certainty, whereas those low on arousal will seek more stimulating environments. Berlyne’s theory has received mixed support because it has poor predictive validity; it is not possible to determine the point of the cusp. There is also some suggestion that when familiarity for an image is controlled for, the relationship between beauty and visual complexity is much more linear in nature (Forsythe et al., 2011).

The competing tensions between beauty and visual complexity perhaps generate some degree of arousal between the existing and the unexpected. We also know that repeated exposure is sufficient to enhance positive attitudes (Zajonc, 1968); perhaps because people are uncertain about how to deal with objects that are novel, repeated exposure acts to make the stimulus more accessible to the individual. Increases in preference are possible with even the slightest repeated exposure, hence the term mere exposure effect (meaning that the object is just accessible to perception). As with the beauty and visual complexity, mere exposure is subject to an inverted U-shaped relationship. Whilst preference increases initially with exposure to a stimulus, later repeated exposure elicits a decrease in preference (Moreland & Topolinski, 2010). Bornstein (1989) attributes this effect to “attributional discounting.” Liking increases with repeated exposure, but observers attribute some of this liking to the exposure processes: *I have seen it often therefore I like it because it is familiar to me*. If, however, observers are not aware of repeated exposure to a stimulus, the discounting effect does not occur. When exposure frequencies and familiarity ratings were used to predict preference, each variable contributed differently to preference judgments and suggesting that the mere exposure effect could take place without learning occurring (Moreland & Zajonc, 1977; Moreland & Zajonic, 1979), suggesting that exposure contributes to preference regardless to how familiar an object is.

Although psychologists have known for some time that preference and exposure are related (Fechner, 1876), the mere exposure phenomenon is still of significant interest to the field. Contemporary research is linking exposure to perceptual fluency or the speed to which a stimulus is processed and greater perceptual fluency generates a positive affect (see Moreland & Topolinski, 2010, for a review). With this in mind we offer new ratings for the Rossion and Pourtois picture sets (line drawings, gray-scale and colorized images) for beauty and exposure to these pictures. Exposure is operationalized by self-report measures of how often participants “encounter” the images. Such measures have not been collected to date and the work of Moreland and Zajonc suggest that encountering something on a regular or irregular basis may not necessarily be the same in concept as being familiar with an image, and that familiarity and exposure may contribute to judgments of beauty in different ways. We may be familiar with what a butterfly is, but we do not necessarily encounter the insect every day. Encounter is a variable, which perhaps captures exposure effects and could be potentially useful to researchers when attempting to measure preferences for pictures.

## Method

### Participants

Eleven different groups of 30 participants from three UK University student populations (n = 330) took part in this experiment. For ten of the groups participants rated only one image construct for one image type (colorised, gray-scale, or line drawing). Rossion and Pourtois (2004) collected their data set from French-speaking students. It could be argued that any differences in data sets could be due to cultural factors. To determine if this were the case, as a control, ratings were collected from one group (n = 30) for both complexity and familiarity simultaneously, with the aim of determining if any cultural differences existed between the French and UK populations.

### Stimuli

The Rossion and Pourtois (2004) image sets for colorised, gray-scale, and line drawings were presented in a PowerPoint presentation on a screen resolution of 1,024 × 768. In the Rossion and Pourtois (R&P) methodology, each stimulus was preceded by an attention signal (!) for 500 ms and, after a brief blank screen (150 ms), was presented for 3,000 ms; however, because participants rated for two constructs they were exposed for 6,000 ms in total. For complete comparability of data, our participants viewed each image for 6,000 ms.

### Procedure

Participants were asked to rate pictures on a Likert scale from 1–5. Ratings were collected for the variables of complexity, familiarity, and encounter for colorised, gray-scale, and line drawings. A score of 1 represented a picture that was not at all familiar; a score of 5 was an image that was very familiar. For encounter, participants were asked to consider how often they encountered the items in the pictures. A score of 1 was not very often and a score of 5 was very often. Complexity was described as the “amount of detail or intricacy” (Snodgrass & Vanderwart, 1980) in the image with a score of 1 being very simple and a score of 5 being very complex.

An additional set of ratings was obtained for beauty for the line-drawing picture set. Participants were asked to consider on a 5-point scale the extent to which something was considered to be beautiful (“not at all” or “very much”). Norms were not obtained for the colorised or gray-scale sets as it was considered that color and shading could act as mediating factors in judgments of beauty.

### Automated measures

The R&P picture sets (n = 260) were analyzed using the two most reliable compression measures as possible automated measures of visual complexity (Forsythe et al., 2011). Jpeg (lossy compression) is a technique that reduces the size of the image file by removing redundant information, but generally assumes that some loss of information is acceptable; this means that Jpeg compression does not always reconstruct an image to its original format and is susceptible to the inclusion of compression artefacts also known as pixilation. Gif (lossless compression) works on a similar principle except that when the image is to be recovered no image loss occurs; for this reason it works well in compressing images that have sharp transitions such as diagrams, text, or line drawings. However, because Gif retains more of the integrity of the image, Gif can only compress to 50 % of the image size. Here images were compressed both in GIF and in JPEG to a 50 % compression size.

## Results

### Analysis 1: Rossion and Pourtsis contrasted with image constructs collected in isolation

Table 1 shows the means, standard deviations, kurtosis, skew and errors for each of the automated and human counts of complexity, familiarity, and encounter. Subjective judgments of familiarity show evidence of skew and on histogram inspection it is apparent that for the Rossion and Pourtois picture set participants perceive a large number of very familiar images. For the line drawing set, no images received mean ratings below the mid-point of 3, for gray-scale and color ratings started at point 2.

**Table 1:** Summary statistics for the three image sets

(n = 260)	Complexity		Familiarity		Encounter	Gif	JPEG	Beauty
	Forsythe	R&P	Forsythe	R&P				
Line								
Mean	2.66	2.77	4.17	3.59	3.33	3,140.84	2,169.18	3.15
SD	.85	1.03	.55	1.01	1.13	943.24	420.98	.63
Skew	.15	.11	−.48	−.32	−.28	.76	.52	.86
Skew error	.15	.15	.15	.15	.15	.15	.15	.15
Kurtosis	−.74	−1.02	−.70	−1.04	−1.24	.51	.21	−.67
Kurtosis error	.30	.30	.30	.30	.30	.30	.30	.30
Minimum	1.04	1.00	2.72	1.06	1.07	999	1698	1.74
Maximum	4.60	4.82	5.00	5.00	4.97	7,285	33,056	4.66
Gray scale								
Mean	2.65	2.89	2.76	3.52	3.17	4,738.21	1,992.17	
SD	.67	1.03	1.50	.94	.57	1,408.04	352.89	
Skew	−.09	−.02	.22	−.31	.69	.22	.74	
Skew error	.15	.15	.15	.15	.15	.15	.15	
Kurtosis	−.68	−1.12	−1.39	−1.04	.35	−.43	.87	
Kurtosis error	.30	.30	.30	.30	.30	.30	.30	
Minimum	1.09	1.06	1.83	1.41	2.07	1,698	1,326	
Maximum	4.17	4.88	4.96	5.00	4.89	33,056	7,193	
Color								
Mean	2.61	2.01	3.59	3.43	2.79	4,781.22	2,083.27	
SD	.77	.94	.83	1.01	1.18	1,412.77	359.51	
Skew	.14	.21	−.23	−.15	.28	.23	.80	
Skew error	.15	.15	.15	.15	.15	.15	.15	
Kurtosis	−.73	−1.11	−1.10	−1.31	−1.26	−.46	1.16	
Kurtosis error	.30	.30	.30	.30	.30	.30	.30	
Minimum	1.04	1.00	1.73	1.53	1.11	1,811	1,433	
Maximum	4.46	4.65	5.42	5.00	5.00	29,859	8,389	

*SD* standard deviation

For visual complexity, no statistically cross-cultural differences were identified between the UK group and the French group of participants for ratings of complexity and familiarity collected simultaneously. For familiarity, there were no statistically significant differences between the R&P data set and UK data collected simultaneously, suggesting again that any differences in collected norms will not be due to cross-cultural differences.

**Fig. 3:**
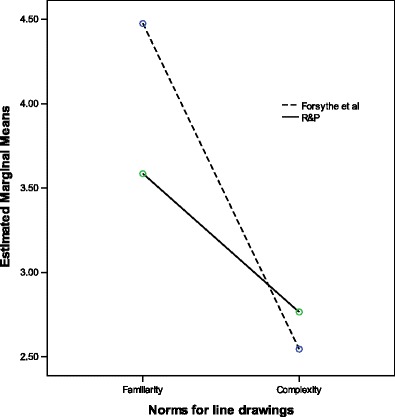
Line drawings mean responses across groups

**Fig. 4:**
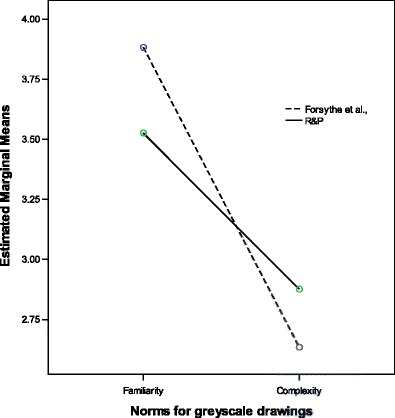
Gray scale mean responses across groups

The differences between the R&P data set and the new norm data reported here were examined using analysis of variance (GLM). There are significant differences between the R&P ratings and new ratings reported here, with large effect sizes across the familiarity and complexity image categories for Line drawings, F(1,518) = 486.18, p < .01, *η*_*p*_^*2*^ .47, Colorized drawings F(1,518) = 157.26, p < .01, η^2^.23, and Gray scale, F(1,518) 160.96, p < .01, η^2^.24. In addition to the main effect, significant interactions were found between the picture sets (Forsythe et al., & R&P) and the norm scores (Figs. 3 and 4) for Line drawings, F(1,518) = 74.89, p < .01, *η*_*p*_^*2*^ .13 and for Gray-scale drawings F(1,518) 9.20, p < .01. *η*_*p*_^*2*^ .02. When compared to the R&P data sets, mean familiarity ratings across data sets for line drawing and gray scale are higher, where as the mean complexity ratings are lower. This difference seems to be less pronounced for colorized drawings.

### Analysis 2: Correlations between image constructs and automated complexity

Before the following correlations were calculated, six outliers were removed on visual inspection of stem and leaf plots and the outlier labelling rule from across the three image sets. These outliers related to images that had unusually large compression scores relative to the remainder of the data set. Table 2 details the correlations between the different variables. For the Line drawing set, the correlation between Gif compression and human judgments of complexity is r_s_.78, p < .01, and for Gif compression r_s_.67, p < .00. These small differences are explained by the lossless technique favored by Gif compression, a method that works best with images that have sharp transitions. Jpeg is known to perform better on images that have high colorization, and this finding is supported by the larger correlate (r_s_ = .61, p < .01). For the Gray scale set there seems to be little difference between the two correlations. Table 2 also demonstrates that measuring visual complexity with compression techniques produces scores that do not correlate strongly with judgments of familiarity.

**Table 2:** Significant Spearman correlations

	Complexity	Familiarity	Encounter
Line			
Familiarity	−.46	1.00	
Encounter	−.48	−.87	1.00
Gif	.78	−.29	−.29
JPeg	.67	−.25	−.25
Gray scale			
Familiarity	−.42	1.00	
Encounter	.32	−.79	1.00
Gif	.55	−.15	_ns_ −.04
JPeg	.58	−.24	_ns_ .00
Color			
Familiarity	−.44	1.00	
Encounter	.47	−.89	1.00
Gif	.54	_ns_−.17	_ns_.14
JPeg	.61	−.24	.21

### Analysis 3: Beauty, exposure, and visual complexity

**Fig. 5:**
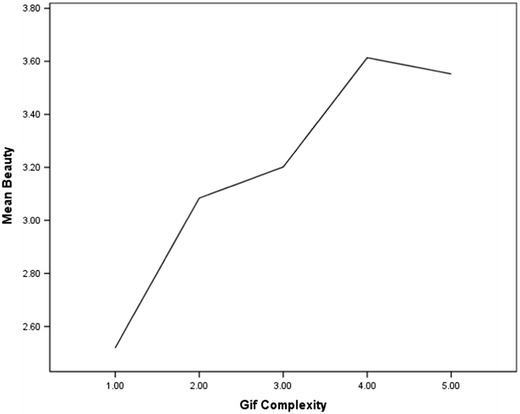
Human judgments of beauty, contrasted with computerized measures of complexity

**Fig. 6:**
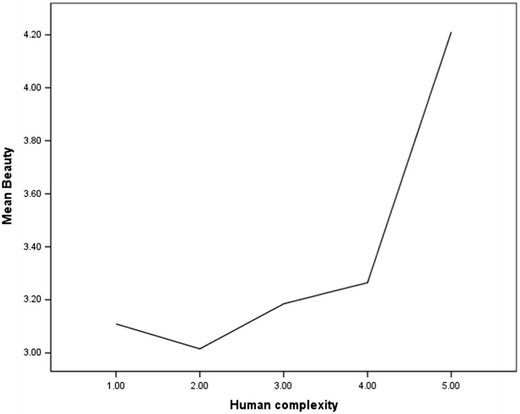
Human judgments of beauty, contrasted with human judgments of complexity

When automated measures for visual complexity are applied there seems to be limited evidence for an inverted U-shaped relationship between complexity and beauty (Figs. 5 and 6). The computerized measures suggest that there is a much sharper climb in preference for images that are above the mean point of visual complexity.

### Predictors of visual complexity and beauty in human judgments

The variables Gif, beauty, familiarity, and encounter were regressed onto the dependent variable human judgments of visual complexity. High correlations between the variables familiarity and encounter contributed to high co-linearity within the model. The model was revised with encounter and familiarity entered separately. The overall model accounted for a moderate percentage of the variance in human judgments of visual complexity (r_2_.62) F(3,256)138.15, p < .01 with Gif emerging as the largest individual predictor variable (β.66, t = 16.25, p < .01), followed by encounter (β − .31, t = -7.52, p < .01). For the revised model, which included familiarity rather than encounter, the overall model variance remained the same. Similar to the encounter variable, familiarity makes a negative individual contribution (β−.29, t = −7.11, p < .01). Scatterplot examination confirmed that images that are less familiar or encountered less frequently are also rated as more complex, findings that complement results reported elsewhere (Forsythe et al., 2008). Neither beauty nor familiarity predicted visual complexity.

The individual contributions towards the dependent variable “beauty” were smaller (r_2_.27 (F(3,256) 6.46, p < .01). The significant predictors were Gif complexity (β.23, t = 2.60, p < .01) and the extent to which viewers encountered the images (β−.25, t = 3.64, p < .01). Again, scatterplot examination determined that images that are encountered less often are perceived as less beautiful. Previous research has suggested that complexity is an important factor in beauty. The data reported here suggest Gif complexity contributed in a small way to perceptions of beauty, but that beauty has no significant relationship with human judgments of visual complexity or familiarity with an image.

## Discussion

Rossion and Pourtois (2004) were interested in measuring complexity because of its impact on processing speed. By developing three new sets of images in gray scale, color, and line drawing they were able to collect normative data (naming agreement, familiarity, complexity, and imagery judgments) for images created similar to those of Snodgrass and Vanderwart (1980). Through the provision of two new object data sets, the authors made an important contribution towards studies of object recognition in normal and clinical populations. These ratings were consequentially used to examine reaction and naming times for these pictures. The authors reported some categorical reaction time advantage – that is, some categories tend to be responded to more quickly than others – although this seemed to be mainly a function of diagnostic color in categories, such as fruits/vegetables versus animals. However, because the authors overlooked convention and collected ratings for familiarity and complexity from the same group of participants some of the reported norms may be confounded.

The aim of the study reported here was to examine the extent to which collecting data from different groups of participants would alter the Rossion and Pourtois data for familiarity and complexity. Our results suggest that when ratings are collected from different groups of observers, scores differ from those reported by Rossion and Pourtois. New norms reported here are systematically rated as more familiar and less complex than previously recorded, with large correlations between complexity norms collected in isolation and compression measures of complexity (Table 2).

If a theory of the perception of complexity as mediated by top-down processing is correct, longer exposure, combined with the request for judgments of familiarity, should lead to pictures being judged as *less* complex and *more* familiar than ratings gathered for complexity alone. Here that does not seem to be the case. Complexity ratings collected separate from familiarity presented mean scores that are lower than norms reported by Rossion and Pourtois. Familiarity ratings collected separately from complexity present a higher norm average than Rossion and Pourtois.

For judgments of familiarity, the data reported here have a larger minimum score, for example 2.72 for line drawings, compared with Rossion and Pourtois (1.06). Such a large minimum score suggests that for the line drawing set, in particular, observers did not feel that there were many unfamiliar pictures in the set and such observations were not found to reflect a cross-cultural effect. Asking observers to consider scoring an image on two variables increases exposure time and the observer would be able to make a thought-out response in regard to how familiar they are with the object in question and how much detail they could see in the object. Ratings for pictures that have been gathered for visual complexity and familiarity could then lead to judgments that are more complex than ratings gathered in isolation. In the original Rossion and Pourtois study each image had an exposure time of 3,000 ms. If participants were to view the image twice then exposure time would increase to 6,000 ms. Time then could have facilitated greater consideration of detail and complexity; however, in the study reported here all images were presented for 6,000 ms, suggesting that his explanation could not hold. A more likely explanation is that perhaps observers became confused. Having rated 260 images for one image construct very quickly (3,000 ms), Rossion and Pourtois required participants to repeat the activity again for a different image construct. Fatigue and interference could have influenced the results, with observers inadvertently rating the images for the wrong image construct or simply becoming bored with the activity. This would explain the unusual anomalies in the distributions of scores between the new data reported her and the R&P image sets, particularly for ratings of visual complexity.

### Beauty, exposure, and visual complexity

The second aim of this study was to further examine the relationship between beauty, exposure to an image, and visual complexity. Data reported here suggests a more linear relationship between judgments of beauty and complexity, a relationship that is somewhat different from the predictions of Berlyne who argued for an inverted U-shaped relationship. A much sharper climb in preference for images that are above the mean point of visual complexity is evident (Figs. 5 and 6).

Whilst the high correlations between the variables encounter and familiarity would suggest they are to some degree tapping the same image constructs, regression analysis presented the encounter variable as explaining more of the variance both in judgments of visual complexity and judgments of beauty. Overall our model explains 62 % of the variance in human judgments of visual complexity, with GIF compression emerging as the largest predictor variable and “encounter” emerging as a marginally stronger predictor variable than familiarity. It would seem then that the number of times in which we encounter an object is a good predictor of human judgments of visual complexity, with images encountered less often being considered more complex. The difference between the two constructs builds on previous research (Forsythe et al., 2008) that suggests that repeated exposure to shapes with meaningless content (i.e., no semantic information), and therefore completely unfamiliar, can reduce perceived visual complexity.

Previous research has suggested that complexity is an important factor in beauty, but our model only predicted a moderate amount of the variance; with compression complexity and the extent to which participants encountered the image emerging as significant individual contributors. Familiarity with the image did not predict beauty, nor did human judgments of complexity. Taken together, these analyses suggest that continuing to collect complexity judgments based on human ratings is a pointless exercise, and that researchers should consider further analysis of the extent to which participants are exposed to, meet with, or encounter an image, rather than simply how familiar the subject mater is.

## Conclusions

The relationship between complexity and familiarity resurrects an old argument that complexity is meaningless; it is the way in which a stimulus is perceived that is important, not the number of elements (Rump, 1968). If complexity correlates negatively with familiarity is it intrinsically bad? If familiarity is a part of the construct of complexity, then this is what researchers and interface designers may need to take into consideration rather than simply arriving at the best, context-free measure of visual complexity. Compression techniques offer researchers the most reliable and user-friendly option for the quantification of visual complexity, they are also unbiased – they are not affected by familiarity with an image set. These metrics have a strong theoretical basis (information theory), produce good approximations of human judgments, and have been demonstrated here as being able to predict human behavior. However, it is a reality that visual complexity is related to familiarity and researchers should consider what it is that they want from a measure of visual complexity and if removing familiarity from the equation is warranted. With this in mind, the reported statistics by Rossion and Pourtois are not intrinsically incorrect; they are simply an alternative way by which to measure picture constructs.

Our findings also determine that perhaps considering the extent to which a person actually encounters an object on a day-to-day basis may be a useful image construct. Encounter seems to explain some of the variance in how people reach judgments of image complexity and in how esthetically pleasing one finds an object.
